# Healthcare Professionals’ Experience of Performing Digital Care Visits—A Scoping Review

**DOI:** 10.3390/life12060913

**Published:** 2022-06-17

**Authors:** Ieva Lampickienė, Nadia Davoody

**Affiliations:** Health Informatics Centre, Department of Learning, Informatics, Management and Ethics, Karolinska Institute, Tomtebodavägen 18A, 171 77 Solna, Sweden; ieva.lampickiene@gmail.com

**Keywords:** digital care visit, online consultation, medical staff, healthcare personnel, user experience

## Abstract

The use of digital care visits has been increasing during the COVID-19 pandemic. Learning more about healthcare professionals’ technology experiences provides valuable insight and a basis for improving digital visits. This study aimed to explore the existing literature on healthcare professionals’ experience performing digital care visits. A scoping review was performed following Arksey & O’Malley’s proposed framework using the Preferred Reporting Items for Systematic reviews and Meta-Analyses. The collected data were analyzed using thematic content analysis. Five main themes were identified in the literature: positive experiences/benefits, facilitators, negative experiences/challenges, barriers, and suggestions for improvement. Healthcare professionals mostly reported having an overall positive experience with digital visits and discovered benefits for themselves and the patients. However, opinions were mixed or negative regarding the complexity of decision making, workload and workflow, suitability of this type of care, and other challenges. The suggestions for improvement included training and education, improvements within the system and tools, along with support for professionals. Despite overall positive experiences and benefits for both professionals and patients, clinicians reported challenges such as physical barriers, technical issues, suitability concerns, and others. Digital care visits could not fully replace face-to-face visits.

## 1. Introduction

Currently, Information and Communications Technology (ICT) plays a significant role in all industries and people’s everyday lives. The healthcare field is no exception. Medical institutions have been using advanced ICT for health records, telemedicine, various forms of e-learning, as well as other tools. The increasing accessibility to the internet and smart devices has influenced the use of applications and implementation of telemedicine in healthcare [1].

One of the concepts used in today’s health care is video-conferencing [2]. Videoconferencing is often described in different terms, such as video meetings [3], digital/virtual meetings, digital visits [4], or video teleconferencing [5]. The concept is rather broad. It includes consultations not only between patients and healthcare professionals [6] but also consultations between two or more healthcare professionals. In which a patient and a healthcare professional are present, on a clinical site or in the home, and together they are consulted by an included specialist from another clinical site [7,8]. So, the consultation may be referred to as video-conferencing [9,10], even though it is broader than just patient-to-healthcare professional consultations. In this review, a narrower concept of video-conferencing is considered central, which is video consultations initiated by patients consulted by health care professionals. This type of consultation is referred to differently in the literature; virtual visits [11], telehealth which can mean both telephone and video consultations [12,13], digital visits, or video consultations [13], to name a few. 

It is important to note that the use of video visits has increased due to both its advantages, such as providing timely care to patients in rural areas or homebound chronically ill patients, and the occurrence of the COVID-19 pandemic. While the pandemic has not been the only factor driving the adoption of digital care visits, it still played a significant role in the process. Due to the widespread infection, most countries implemented public restrictions and recommendations such as minimizing or banning gatherings, countrywide lockdowns, social distancing, wearing protective masks, and paying special attention to hand hygiene to control the contagion [14]. The infection rates increased exponentially during the first and second waves of the pandemic, and there were large numbers of severely ill patients that needed immediate hospitalization and even intensive care [14]. This resulted in an unusually high workload for the healthcare sector; multiple hospital wards were transformed into COVID-19 wards due to the shortage of beds in intensive care units [15,16,17]. The situation was so severe that routine visits and other non-emergency procedures had to be postponed, prioritizing COVID-19 patients [14,17].

On account of the circumstances, health care institutions were required to rapidly adopt and implement digital care visits in their practice to be able to provide telemedicine services [18,19]. The urgency of the situation sped up the process of authorization and regulation regarding legal matters such as new payment models for remote health care services and health information privacy [19]. Digital care visits got implemented in various areas of health care—primary care, mental health [20,21], orthopedic care [22], neurology [18,23,24], palliative care [25], pharmacy [26], dentistry [27], and others. Even though digital care visits do not provide possibilities for physical examinations where a healthcare professional would need to examine a patient physically, video consultations allow specialists to evaluate and sometimes diagnose by inspecting the patient through video. The pandemic has brought massive challenges and burdens to this world. Still, it also stimulated people to adapt and seek quick and creative solutions, speeding up technology implementation in different areas, including the health care sector.

Some research has been done regarding the use of video conferencing, implementation issues, policies, etc. [11], along with patients’ experiences and perceptions of using video-conferencing for healthcare visits [6,28,29,30]. However, the number of studies on healthcare professionals’ experience with patient-initiated digital visits is limited.

A broader overview, including healthcare professionals from different specializations and the latest literature, could contribute to a better understanding of what is known on this topic, what the research gaps are, and what should be studied more. Finding out what the benefits and challenges of using digital care visits are from the healthcare professionals’ perspective could help optimize the service for both health workers and patients. Thus making it safer and more usable, resulting in higher satisfaction with the service as well as more efficient use of limited healthcare staff resources.

### Aim

The aim of this study is to explore the existing literature concerning the user experience of digital care visits from different healthcare professionals’ points of view.

## 2. Materials and Methods

A scoping review design was chosen for this study. The review was conducted using the methodological framework by H. Arksey and L. O’Malley [31] and adapted PRISMA-ScR checklist by Tricco et al. [32]. Scoping reviews are “a type of knowledge synthesis, follow a systematic approach to map evidence on a topic and identify main concepts, theories, sources and knowledge gaps” according to A.C.Tricco et al. [32]. This type of review may vary in the breadth of the literature coverage and the depth of the information elicited from it [32].

### 2.1. Search Strategy and Timeframe

Specific search terms and their combinations for finding the literature were thoroughly researched and tested. The search terms were chosen based on the aim of this study and were adjusted to retrieve the most relevant studies that fall under the scope of the selected topic. MeSH term “telemedicine” was used in the test searches and retrieved a large number of results, out of which many were irrelevant as there were publications on phone consultations, remote monitoring, wearable tracking/monitoring devices, etc. Therefore, to narrow down the search and retrieve more relevant results, this term was not used and was replaced with more specific keywords. In addition, the queries were adapted to match each chosen database’s syntax. Three databases were chosen for the search: PubMed, Web of Science, and IEEE Xplore. The search queries used for the selected databases are presented in Table 1. Special tags and MeSH terms were used for targeting the most relevant studies—tag TIAB was used in PubMed for searching in the title, abstract, and keywords, MH for MeSH terms, TS for searching in the title, abstract, and keywords in Web of Science, and “All metadata” for searching in IEEE Xplory. IEEE Xplory digital library does not use MeSH terms. Thus, additional synonyms to some keywords were added to expand the search. The filters applied for the searches were 10 years’ time span, English language, and full text available.

Apart from the database search, grey literature (“includes a range of documents not controlled by commercial publishing organizations”) [33] was searched using similar search terms through Google Scholar and reviewing the reference lists of included studies to identify literature that has not been formally published in scientific journals. Manuscripts that were not yet published, conference papers, dissertations, government documents, and other types of grey literature [34] were searched and screened for eligibility. The search was carried out from 1 March 2021–15 April 2021.

### 2.2. Study Selection

The literature was screened for eligibility based on inclusion and exclusion criteria. The inclusion criteria were original articles, conference proceedings, review articles, and reports published within the last 10 years in English, focused on healthcare professionals’ experience using digital care visits for patient consultations. Papers that fell under the scope and were published within a specified time frame and were retrieved during the “grey literature” search were also included. The exclusion criteria were articles published in languages other than English and earlier than 2011. They focused on patients’ experiences using digital care visits or covering healthcare professionals’ willingness to use digital care visits rather than their experience using it.

**Figure 1:**
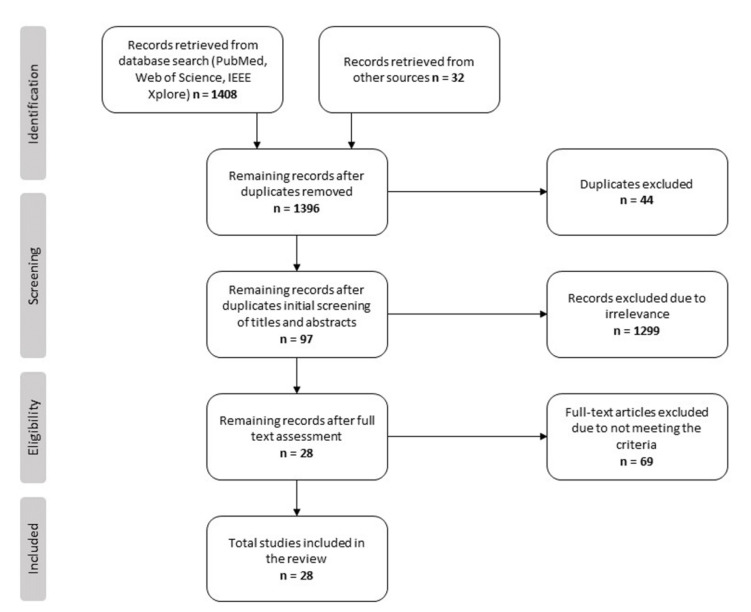
The process of study selection—Preferred Reporting Items for Systematic reviews and Meta-Analyses (PRISMA) flow chart.

For this review, 1440 studies were retrieved and 44 duplicates were removed—more detailed numbers can be found in the flowchart (Figure 1). Citations were handled using the referencing program Mendeley. The initial screening was performed by reading the titles and abstracts of the retrieved results. After the screening, 97 studies were read in full to decide which to include in the review. Out of those 97, 28 studies met the inclusion criteria and were deemed eligible for this study. Studies were excluded if they focused on remote consultations via phone, asynchronous telemedicine using store and forward technology, clinician attitudes towards telemedicine or willingness to use it, healthcare professionals’ experience of using telemedicine for professional-to-professional consultations, or remote monitoring. Studies that explored healthcare professionals’ and patients’ or caregivers’ experience with digital care visits were included if separating clinicians’ experience from the results was possible. Papers in which healthcare professionals’ experience using several methods for providing telemedicine were studied and deemed eligible for the review if it was possible to separate the experience from digital care visits.

### 2.3. Data Analysis

The 28 studies were read again and the information was charted in an MS Excel spreadsheet. Details such as title, publication date, study design, the type of healthcare professionals who participated in the study, country/region, main findings, and other relevant information were documented in the spreadsheet. The emergent themes are presented in the results.

The collected information was analyzed using thematic content analysis. Several themes had emerged, including positive experiences/advantages, facilitators, negative experiences/challenges, barriers, and possible improvements in using digital care visits from healthcare professionals’ experiences. The themes were divided into categories and sub-categories.

### 2.4. Ethical Considerations

Ethical issues were considered for this study, although no human subjects were involved due to the nature of this study. The data analyzed in this review is from published articles and reports that are freely or institutionally accessible. No sensitive data such as real medical records were used, meaning that no one’s integrity was compromised.

## 3. Results

### 3.1. General Characteristics of the Reviewed Studies

Most of the selected studies were published in the last 5 years, while only one study was published earlier in 2015. More than two-thirds of the included papers were recent and published in 2020 or 2021. The studies were carried out in different countries, Australia (n = 4) and Europe (n = 10), while half of the studies originated from the United States of America (n = 14). More than a half of the studies explored the experiences of physicians, among which were medical oncology professionals [35,36], general practitioners [13,37,38,39], otolaryngologists [40], urologists [41], cardiologists [42], and sports medicine professionals (physiatrists) [43,44]. Another considerable group of professionals was mental health professionals—therapists and psychotherapists—who participated in eight studies. The experiences of other healthcare professionals such as nurses, advanced practice professionals, dieticians, and physical therapists were studied in nine papers. Non-medical professionals, patients, and caregivers were included in some studies; however, their experiences were separated in the results, and findings regarding their experience were not included in this review. Fifteen studies were related to the ongoing COVID-19 pandemic (Table 2).

Five major themes emerged from the data—positive experiences/benefits, facilitators, negative experiences/challenges, barriers, and possible improvements in digital care visits. Each of these themes had multiple categories and sub-categories. The categories will be used as subheadings further on. The results of each will be presented and explained in more detail. (Table 3, Table 4 and Table 5).

### 3.2. Positive Experiences/Benefits of Digital Care Visits

Numerous benefits and aspects of a positive experience have been reported in the reviewed literature. Aspects such as benefits of remote work, efficiency, satisfaction with digital care visits, and benefits for the patient were identified (Table 3).

#### 3.2.1. Benefits of Remote Work

Flexibility regarding working hours and the workplace has repeatedly been reported in the literature. Cioffi’s study has found that nearly 60% of psychotherapists reported greater flexibility [54]. In Koch and Guhres’s research, physicians expressed that digital care visits allow “flexibility to work from home” and “flexibility regarding working time” [13]. Hardy et al., in their mixed-methods study, found that therapists providing teletherapy for couples felt similarly “Flexibility in scheduling and location” [45]. Björndell & Premberg have found that “the flexibility of work and the regulated assignment online were positive for the physicians’ work situation and well-being” [39]. Sugarman et al. have also discovered that therapists identify “scheduling/flexibility” as an advantage [46]. The same findings mentioned in Fernemark et al. study—flexibility with work hours and the ability to choose where to work from, were considered advantages by general practitioners in Sweden [37].

Less travel time and costs were also mentioned as benefits in several publications. Physicians from three studies think the use of digital care visits saves commuting time [39,41,46]. This plays a role in enhancing healthcare professionals’ quality of life: “saving travel time, being present at home, and participating in family activities, etc., was considered beneficial” [39].

Digital care visits provide flexibility which contributes to healthcare professionals feeling less stressed and more at ease. A small percentage of psychotherapists in an Italian study agreed that they felt more relaxed during online sessions [54]. Björndell & Premberg wrote that “working from home was appreciated by the physicians because it let them work in peace, feel less stressed, and enjoy being at home” [39]. 

Sugarman et al. reported that digital care visit “supports personal safety concerns, including COVID-19 risk” [46], and Björndell & Premberg mentioned “reduced risk of infection transmission” in their paper [39].

#### 3.2.2. Efficiency

It was indicated in several studies that working with digital care visits is more efficient in the sense that it saves time and increases productivity. Kemp et al. [47], Koch & Guhres [13], as well as Saiyed et al. [48] studies have shown that digital visits took less time than in-person visits, according to healthcare professionals. A total of eight of twenty-eight selected studies indicated that digital care visits increase productivity or efficiency. Healthcare professionals felt that by using telemedicine, they could be more productive [41,54], more structured and efficient due to greater focus during the sessions [49]. The use of technology made the visits more efficient [35], meaning that the patients were prepared, and physicians could easily end the video calls after the consultation and consult another patient right away [47,59]. Some physicians reported that it was easier to consult patients via digital care visits as their cases were simpler than those in the physical visits, making it possible to provide consultation to more patients [37,39].

#### 3.2.3. Satisfaction

Overall, healthcare professionals, regardless of specialization or location, had mostly positive experiences with digital care visits, ranging from at least slightly [45] to highly satisfied [35,40,44], as stated in nearly half of the selected publications. Professionals felt that digital care visits have a positive impact on their work environment [13], were generally happy with their experience [41,43,55,58,59], and enjoyed it [48].

The usability of digital visits varied due to the use of different platforms. Some studies stated that systems used for digital care visits were easy [41,44], and twelve out of twenty-eight studies found that they were quite straightforward and easy to use [13,39,41,43,46,48,50,55,59,60,61]. Several publications revealed that healthcare professionals were satisfied with the system/platform itself [13,47,58] and/or appreciated its features [46]. 

Regarding the interaction between healthcare professionals and patients, healthcare professionals in six studies expressed having a positive experience regarding patient-professional interaction. Clinicians could discuss patients’ issues, assess their condition, and offer treatment advice effectively. In many cases, an in-person visit was unnecessary [39,43,48,55,58].

#### 3.2.4. Convenient, Accessible Care and Saved Resources for Patients

Increased flexibility and greater accessibility are some of the benefits of digital care visits. Digital care visits allow patients to schedule visits at their convenience [13,39,46,58,59]. Patients with responsibilities for, e.g., caring for their children, do not need to organize childcare for visiting healthcare professionals [46,51,52,57]. Digital care visits offer high-quality medical care for patients from rural or remote areas where such care is inaccessible [13,39,41,52,58]. In addition, patients who are homebound due to their medical conditions or those who simply do not have the means or wish to travel to a health care facility benefit from having their visit digitally [46,51,57,59]. Saving travel costs was mentioned multiple times in eight reviewed articles [13,46,51,52,57,58,59]. Moreover, patients feel more at ease when they are surrounded by the environment and people that they are used to, such as their family or their pets [39,45,58,59]. Digital care visits were emphasized in mental health-related studies as well. Studies showed that patients tended to be more open, feel more secure, and often shared more with their therapists during the online sessions from their homes [45,46,49]. Having remote visits from patients’ homes allows for better family member inclusion [51], knowing more about their condition, and caring for them [51]. Hinman et al. study on remote physiotherapy found that patients felt empowered when doing exercises at home. Digital care visits increased their adherence to the program and allowed them to learn correct and safe exercise techniques [59]. Patients gained more confidence in performing rehabilitation exercises at home [59] and took more initiative to care for themselves and be more self-reliant [60]. They even could form stronger therapeutic alliances or cooperation with therapists [45,46].

Furthermore, by having remote health care visits, patients avoided transmission of and exposure to communicable diseases, which enhanced their safety and contributed to controlling the spread of infectious diseases [39,51]. Overall, clinicians from several studies indicated they felt their patients were satisfied with digital care visits, their complaints were addressed, and they got the necessary care [37,38,48,56].

### 3.3. Facilitators

#### 3.3.1. New Perspectives in Remote Care

Digital care visits employ video-conferencing technology, opening new perspectives in remote care. The ability to get instant non-verbal feedback through video, i.e., seeing the patients’ facial reactions and body language, enables healthcare professionals to get more unspoken information from the visit [59]. Seeing a patient’s symptoms during the video consultation allows health care professionals to intervene in real-time [57]. Some clinicians thought that caring for patients remotely made them focus more on what was the most important in the treatment [49,59].

Clinicians noted that digital care visits felt more personal in Hinman’s study because physical therapists had to listen to their patients to provide good service [59]. Levy et al. stated that in a therapeutic setting with a close-up video of the patient’s face, the session could be as intimate as in-person [52]. Being able to see the patient was one of the reasons for healthcare professionals’ preference for digital care visits over phone consultations [13,60]. Interestingly, another new perspective brought by digital care visits, which was not present in traditional visits, is a possibility to get insight into a patient’s home environment. This allows clinicians to get a better overview of the patient’s life and gives valuable insight into how they communicate, e.g., with their relatives or pets if they are in the picture, which creates a unique possibility to “get closer” to the patient and many healthcare professionals appreciated that [35,39,45,46,57]. According to clinicians, digital care visits allow for shorter and more frequent visits [46,49,51] and ensure continuity of care [45,46], supporting access to care for patients.

#### 3.3.2. Technical Qualities

Eight out of twenty-eight studies indicated that technical features such as audio and video were of good quality or that there were no issues during the visits [13,37,38,43,48,55,59,61]. Clinicians thought they could hear and see patients well enough to provide healthcare services.

#### 3.3.3. Possibilities of Digital Care Visits

Using video-conferencing technology for digital care visits, it is possible to consult, examine, and diagnose patients, as stated in eight reviewed studies [35,39,43,45,48,57,58,61]. Digital care visits seemed like a suitable form of care for some clinicians [58]. A total of 57% of therapists in Becevic’s et al. study reported that they could treat patients via digital care visits [61]. Furthermore, 83% of couple therapists in Hardy et al. study replied that they could at least somewhat solve the conflicts as effectively as in in-person visits [45]. In the Kirby et al. survey, surgeons were fairly confident in their diagnoses and assessments [43]. Other studies showed that clinicians were comfortable treating their patients or that their patients were appropriate subjects for getting treatment via telemedicine [35,39,48]. Several studies indicated that clinicians felt they could establish a connection with patients, an imperative part of patient-clinician interaction [45,46]. Some even stated that the relationship with patients was as authentic as face-to-face visits [48,56]. 

#### 3.3.4. Suitability

A total of four of the selected studies explained that digital care visits are best suitable for follow-up visits, as it is easier to consult a patient who is known and whose condition is not completely new for the healthcare professional [38,40,44,55]. Some other studies showed that, in clinicians’ opinion, digital care visits are suitable for treating mental health problems as no physical examination is required [38,39]. In addition, digital visits are appropriate for some less complicated skin conditions if the video quality is good enough [39,46]. This type of visit is also suitable for palliative and pediatric care [35], for chronic disease management [40], and for administrative purposes such as extending a sick leave for working patients [38].

### 3.4. Negative Experiences/Challenges of Using Digital Care Visits

Despite numerous advantages of digital care visits, multiple drawbacks and challenges are reported in the literature. Clinicians have encountered decision-making issues, workload and workflow problems, patient-professional relationship-related considerations, patient-related challenges, or low satisfaction. These negative experiences and challenges will be presented further on. 

#### 3.4.1. Complicated Decision Making

Seven studies out of twenty-eight declared that clinicians experience difficulties making decisions regarding a patient’s diagnosis, treatment, or referrals [13,37,38,39,46,50,55]. In Koch & Guhres’s paper, physicians reported that “information for decision making is limited” in digital care visits [13]. The Johnsen et al. study revealed that 15% of GPs were worried that they had possibly missed signs of serious disease. In addition, more than half of the physicians considered the inability to perform a physical examination was a serious disadvantage [38]. In another publication, it was explained that physicians think digital care visits will never be able to replace hands-on examination [55]. Sugarman et al. articulated that according to therapists’ experience, it was complicated to easily treat distracted patients, to visualize their psychomotor symptoms, measure vital signs, and prescribe medication based on their observations and discussions during the digital care visit [46]. Other authors got similar findings regarding these difficulties [37,39,51,52,59]. Physicians and therapists also saw disadvantages in having to rely on the patients’ observations and descriptions to diagnose, assess, or prescribe proper treatment [35,39,51,52,59]. GPs were sometimes hesitant about trusting a patient’s complaints without an examination when extending their sick leaves or prescribing medication [39].

Several studies revealed that healthcare professionals had difficulties guiding the right patients to digital care visits. It was complicated for physicians to sort the patients whose conditions were appropriate to be treated via digital care visits, who needed to have an in-person visit, and who could have their problems solved by other health professionals, e.g., by nurses to utilize the limited healthcare resources efficiently [13,37,39].

#### 3.4.2. Professional Development and Work Environment

Clinicians’ concerns, such as lack of medical skills practice and loss of competence, were raised in the Fernemark et al. paper [37]. GPs worried that digital care visits often deal with simpler cases where physical examinations and more complicated medical manipulations are unnecessary. They were concerned that by working exclusively with digital care visits, they would lose some of their skills and competence [37]. 

#### 3.4.3. Workload and Workflow

Clinicians in several studies reported that digital care visits require a higher level of concentration compared to traditional visits [45,54,57]. Over 55% of therapists said digital care visits require a higher concentration level in the Cioffi et al. study [54]. Therapists from another study pointed out that 30% of them experienced less engagement, that they had to work harder because they needed to monitor technical aspects of the session, that it tended to non-verbal communication ( difficult for 80% of the therapists), and that disruptions during the visits occurred for 92% of the respondents [57]. Therapists in the Hardy et al. study experienced “clinician fatigue—lethargy, tiredness, and discomfort” and claimed digital care visits were more tiring [45]. Similarly, other studies reported that treating patients online is more tiring as the clinician needs to compensate for the absence of physical presence, focus harder, and use senses other than touch to assess patients, as well as often helping patients with technology issues and dealing with a sometimes higher workload [45,49,54,56,57]. Healthcare professionals from the Cioffi et al. and Fernemark et al. studies felt it was more difficult to structure their time when working from home, and they were unsure as to when and if they should take breaks [37,54].

Around one-fifth of the physicians who participated in the Gold et al. survey replied that they experience increased stress while working with digital care visits [50]. Some identified that the type of digital care visits conflicted with their views on how care should be delivered [50]. Johnsson et al. and Paulik et al. discovered that clinicians feel administration and preparation for digital care visits takes a lot of time, because they must adapt certain treatment techniques to a new setting [36,49]. Other authors found that clinicians experience a lack of administrative support, and they need to schedule visits and do other tasks, that a secretary or a nurse could take over, instead of focusing on treating patients [35,36,41].

#### 3.4.4. Dissatisfaction

One study from the USA reported that 58% of the participating physicians were generally neutral or dissatisfied with digital care visits. Nearly half were concerned that the healthcare professional-patient relationship was compromised because of digital care visits [53].

Two other studies discovered that clinicians felt their patients’ needs were not adequately addressed as some patients could not get the necessary care online, or wished for a physical presence and social interaction with the clinician [53,60]. Overall, five studies reported that healthcare professionals consider digital care visits inferior to face-to-face visits and prefer traditional visits over digital ones [36,45,50,52,53].

#### 3.4.5. Patient-Professional Relationship

Several studies addressed the issue of the healthcare professional’s difficulty fostering rapport with their patients [44,56]. Bekes et al. and Tenforde et al. reported that healthcare professionals felt they had difficulties connecting emotionally to the patient [44,56]. Mental health professionals expressed that it was difficult to deal with emotional situations in digital care visits [45,49,52,56] mainly due to the inability to properly see patients’ body language and facial expressions and the inability to use certain conflict management techniques from a distance [45,49,52,56].

Another concern regarding the patient-professional relationship was that digital care visits are less personal than face-to-face visits. This concern was reported in two studies that explained it happened due to a lack of physical presence and being there for the patient [52,60]. Similarly, therapists from the Wade et al. study felt that therapy sessions via digital care visits were less intimate [57]. Mental health workers experienced difficulty maintaining patients’ attention and engagement due to their condition or distractions at home [45,46,56,57].

#### 3.4.6. Unmet Patients’ Expectations

A few studies reported that healthcare professionals felt their patients’ desire for physical consultation was unmet, especially oncological and geriatric patients [51,60]. At times, according to clinicians, patients desired to be examined physically to feel more secure regarding their diagnosis [51] or because social interaction was important for homebound patients [60]. Koch & Guhres found that physicians reported patients having unrealistic expectations or poor understanding of what could be done during digital care visits, resulting in dissatisfaction [13].

#### 3.4.7. Technical Challenges

The fact that patients have different socioeconomic statuses was related to their access to technology such as smartphones, tablets, and computers [51]. This meant that not all patients got access to digital care visits. In addition, patients’ lack of technological skills [45,51] or patients’ lack of comfort in using technology [45,49,51,57] often prevented successful interaction via digital care visits. 

#### 3.4.8. Complications from the Patients’ Side

Other challenges clinicians had to deal with were more visit cancellations or rescheduling by patients due to increased flexibility, as reported by Hinman et al. and Wade et al. [57,59]. Disruptions when patients get distracted by their family members or daily chores also had a negative effect on the overall experience [45,57]. Moreover, Heyer et al. stated in their study that sometimes patients do not feel they should pay for digital care visits the same as they do for traditional ones [51]. 

#### 3.4.9. Safety and Privacy

Several studies have covered clinicians’ concerns regarding patient safety, privacy, confidentiality, and informed consent. Patients’ immediate safety due to acute conditions and the need for emergency hospitalization [37,38] or safety regarding conflicts at home and risks posed by their mental state [45] concerned clinicians. Privacy was an issue discussed in three studies. In therapy sessions, privacy is important, and it is severely compromised when a patient is unable to find a place in their homes where they feel secure and cannot be overheard by family members [45,52,56].

### 3.5. Barriers

#### 3.5.1. Physical Barriers

Using digital care visits for treatment sometimes poses barriers, such as the inability to apply certain treatment techniques that could otherwise be used in a face-to-face visit. Cioffi et al. showed that 50.69% of responding therapists felt digital care visits restrict or prevent applying certain techniques [54], and surgeons from Kirby et al. had similar experiences [43]. Allied health specialists reported that the medical interventions were limited to those who did not require a trained occupational therapist’s presence. Therefore, patients were less successful in reaching motor goals [36]. 

A similar problem occurred to healthcare professionals trying to examine the patients. In 13 studies, clinicians reported that lack of physical examination was problematic [36,38,39,43,44,46,48,50,51,55,58,59,61]. Surgeons in Kirby et al. pointed out that they had difficulty measuring sensation and tenderness [43]. In addition, occupational therapists sometimes struggle to evaluate motor skills [36]. Mammen et al. found occasional technical problems and the inability to touch sometimes hindered the physician’s ability to conduct the examination [58]. Physical therapists experienced discomfort without hands-on assessment [59]. Other studies showed that clinicians thought not having a physical examination was a loss, and digital care visits cannot replace hands-on examination [38,39,44,46,48,50,51,55,61]. Mental health workers [45,49,52,57] and physicians [48,50] considered the inability to see non-verbal cues as a disadvantage.

#### 3.5.2. Suitability

Clinicians expressed that digital care visits were not always a suitable form of care for some patients. Studies have found that digital care visits were less applicable for new patients [38,50]. This also applies to patients that have musculoskeletal, skin [38,50], pediatric problems, acute and severe health issues [38,51], or conditions that certainly require physical examination [35]. They were also unsuitable for patients with severe mental problems such as paranoia, psychosis, etc. [46]. Medical oncology professionals noted that digital care visits were inappropriate for sensitive conversations with the patient, such as for delivering bad news [51].

#### 3.5.3. Technical Issues

Technical issues may become a serious barrier to providing quality care. In ten of twenty-eight studies, authors reported that healthcare professionals had encountered connectivity issues. Lost connection [36,45,51,52,60], difficulty logging on [46], patients not being able to connect [42], poor or unstable internet connection [50,60], over half the clinicians in Mammen et al. and Yu et al. indicated they experienced audio, video, and connectivity issues [53,58]. Healthcare professionals from other studies also expressed they had problems with poor quality sound or video during the visits, which affected the overall quality of the consultation as it was more difficult to communicate and assess the patient [42,46,52,60,61].

#### 3.5.4. Reimbursement Issues

Healthcare professionals mentioned problems related to reimbursement for digital care visits. Due to rapid telehealth adoption, the insurance companies have not adapted their policies for coverage regarding digital care visits, which is problematic for healthcare professionals [51]. Allied health therapists experienced they had to spend a lot of time training to use digital care visits and administrate them. At the same time, they were only compensated for the factual duration of the visits, not the preparation, which posed a risk of job dissatisfaction [36]. Other authors suggested the reimbursement models should be adapted to offer fair pay for healthcare professionals providing care via this technology [13,41,44]. Negative experiences and challenges are presented in Table 4.

### 3.6. Suggestions for Improvement

Lastly, a theme about possible suggestions for improving digital visits emerged from the reviewed literature. The findings suggest that main improvements could be done in training and education, improving tools, and adapting the system, as well as offering greater support for clinicians. 

#### 3.6.1. Training and Education 

Providing proper training for healthcare professionals on how to use the technology and train them in providing health care services remotely would be beneficial and improve clinicians’ experience, as well as increase their confidence in using digital care visits, as reported in six studies [13,36,42,44,47,56]. Preparing tutorial materials such as video clips or booklets concerning how to use the digital care visit platform to support both professionals and patients was indicated as a potential benefit [55,59]. Promotion and education about digital care visits could raise awareness and encourage and support healthcare professionals in using the technology [41,44]. 

#### 3.6.2. System and Tools 

Standardized equipment for providers would ensure that digital care visit platforms are supported by all used devices, and it would be easier to use, as stated in Kemp et al. [47]. Clinicians would also benefit from the video-conferencing tool being integrated into the EHR system they use routinely to grant easy access to patient records [37]. Additionally, healthcare professionals find it difficult to guide appropriate patients to digital care; therefore, implementing a triage system would be helpful [13,37,50]. In response to safety and confidentiality concerns, improvements could be made to enhance security by setting session passwords, end-to-end encryption, and ensuring GDPR compliance [42]. Paulik et al. suggested using two cameras for the patients—one showing a close-up image of the face and another capturing the whole body to improve visibility and understanding of non-verbal cues shared by the patient during therapy sessions [49].

#### 3.6.3. Clinician Support

Providing clinicians with properly functioning devices and ensuring they have a suitable work environment that is private, quiet, and well-lit would contribute to the professional’s comfort and the quality of the consultation [45]. Not putting a burden of excessive administration and coordination tasks that could be done by other staff on the clinicians [42,44,47] could help them better cope with the workload. Lastly, another important aspect is the promotion of self-care. It has been reported that digital care visits may be more tiring than regular sessions, and professionals caring for themselves to cope with fatigue caused by digital care visits is crucial [46]. Suggestions for improvement are presented in Table 5.

## 4. Discussion

This study aimed to explore the literature and determine the user experience of digital care visits from different healthcare professionals’ points of view [62]. This study showed that healthcare professionals mostly had positive experiences with digital care visits. Many authors stated that healthcare professionals believe that digital care visits are advantageous for the professionals considering benefits such as remote work, efficiency, satisfaction with this type of consultation, and new perspectives in remote care. Clinicians particularly appreciated the ability to be flexible in terms of work hours, choosing the work environment, increased productivity and efficiency, as well as the ease of use of the technology. Similarly, when exploring patients’ points of view, a systematic review has shown that the patients had overall high satisfaction with information sharing and consumer focus [6].

Many healthcare professionals agreed that digital care visits significantly increased the accessibility of health care services to patients who live in remote locations, are not able to travel to a health facility due to various reasons (limited or restricted mobility, social phobias, lack of resources, etc.,), or even those with responsibilities at home such as caring for young children or sick relatives. These findings align with other studies that explored the caregivers’ and patients’ points of view toward remote care [63,64]. A significant portion of studies declared clinicians found their patients became more confident in themselves, felt more at ease, and cooperated in their treatment better when they had the chance to stay in their home environment. Specifically, this was mentioned not only by mental health professionals, who rarely need to apply hands-on techniques in their treatment, but also by physical therapists who were teaching their patients exercise techniques and managed to achieve good outcomes [39,45,58,59]. Facilitators found in the literature were related to new perspectives and features of remote care, such as real-time video aspects that added visual information compared to consultations over the phone.

Health care professionals had mixed experiences with technical quality. Eight of the selected papers reported the quality as being good with no issues at all. In ten of the other studies, it was reported that clinicians experienced technical issues related to video/audio quality or connectivity issues from their or their patients’ side [13,37,38,43,48,55,59,61].

The possibilities of digital care visits were rated positively among healthcare professionals. Physicians and therapists thought it was possible to consult, examine, diagnose, and treat patients via digital care visits. However, in almost half of the reviewed papers, healthcare professionals expressed that the lack of physical examination was at least somewhat problematic. Particularly, general practitioners and healthcare professionals who work with musculoskeletal disorders and oncologic patients found the inability to physically examine patients to be an obstacle in some cases [36,38,39,43,44,46,48,50,51,55,58,59,61]. Overall, there were mixed opinions on whether digital care visits could replace face-to-face visits. Health care professionals reported that digital care visits are suitable to assess some conditions, such as simpler skin conditions, mental health issues, and other conditions that did not require touch to assess as well as follow-up visits for chronically ill patients [38,39,40,46]. On the other hand, when the conditions were more complicated or the patient was new, clinicians reported that a physical visit would be more suitable [35,38,46,50,51]. Compared with another study, health personnel found both benefits and disadvantages of treating patients remotely. Some found it advantageous because the patients did not need to wait long to receive care; others expressed it was easier for them to write a referral rather than have a digital care visit [64].

The findings suggest that digital care visits are suitable for visits that involve treatment of rather simple conditions. Those that do not require a physical examination or do not involve sensitive conversations would be better managed in face-to-face visits. Naturally, selecting the right kind of patients for remote care would decrease the complexity of decision-making when a professional must rely on other senses and information collected without being physically present with a patient. This could be achieved by employing a triage system as suggested in two Swedish studies [13,37] and one American study [50]. By implementing a triage system that would filter the patients and direct them to the right type of care, limited medical resources could be utilized more efficiently. Other suggestions for improvement include training and educational materials, which could potentially improve healthcare professionals’ experience in using digital care visits, as well as raise awareness among those who have not started to use it yet and encourage them to employ the technology [13,36,42,44,47,56]. Conversely, general practitioners from another study noted that even though reading manuals on how to use the technology were often helpful, they rarely found the time “to read and understand the instructions” due to tight scheduling [64].

Some healthcare professionals expressed digital care visits were not well integrated into their workflow. They felt unsure when prescribing medication to patients without knowing their health history [39]. Using separate video-conferencing tools, scheduling consultations, and coordinating remote care added to the workload [50], thus implementing the necessary tools into the EHR system could make the workflow smoother and allow healthcare professionals to access patients’ health records, ensuring greater confidence for the clinicians and safety for the patient [37]. Another important aspect of remote consultations or remote work, in general, is fatigue that comes from communicating online and the feeling of isolation and loneliness from being unable to meet with peers. One study suggested that self-care should be promoted among healthcare professionals working remotely [46]. Online social activities for the healthcare teams such as communication channels, virtual social groups, peer support, and games or team challenges could be offered to healthcare professionals to provide them with an opportunity for casual and less formal communication with colleagues as a replacement for running into each other at the office [65]. This, in turn, could make them feel more connected to the team and less isolated.

Few of the studies in this review involved resident doctors or young professionals who do not have extensive work experience [50,51,53]. It was reported that they had more difficulties in using digital care visits. It was more complicated to assess and diagnose patients due to limited work experience [50,51,53], and therefore it is possible their experience with remote consultations was more negative. It is possible that clinicians with more in-person work experience would be a better fit for providing such services [39], and they would be more comfortable in such a setting. Alternatively, it could be beneficial if young professionals got mentorship or support from their more experienced peers whenever they needed to increase their confidence. Also, as mentioned in some of the reviewed articles, training and education on how to use the technology and provide remote health care would be beneficial [66].

It is worth mentioning that because of the COVID-19 pandemic, the adoption of digital care visits was extremely rapid, and many health organizations were unprepared for it. Health workers were pushed out of their comfort zones and forced to move to remote care abruptly without having the time to prepare or train for it properly, and many of the organizational changes had to be made suddenly to make the shift happen [45,46,54]. Therefore, the studies published during the pandemic were strongly influenced by these aspects, and healthcare professionals’ experiences of using digital care visits were affected by the sudden shift as well as general stress and pressure caused by this unprecedented contagion. Many of the professionals have not used digital care visits prior to the pandemic, and the sudden change may have influenced their experiences more negatively. However, on the contrary, many stated an overall positive experience and would continue to use the technology to a smaller or larger extent in the post-pandemic future [35,38,41,44,45].

It is clear from the results that digital care visits will never fully replace in-person visits [67,68]. However, the studies showed that it is possible to provide health care services via digital care visits in cases that do not require a physical examination for the assessment, such as chronic disease management [40]. Patients with conditions such as diabetes who are consulted and monitored by healthcare professionals online may be able to manage their condition at home without the need for hospitalization, thus saving time and resources for both parties [68,69,70]. Almathami et al. performed a systematic literature review on “Barriers and facilitators that influence telemedicine-based, real-time, online consultation at patients’ homes” in 2020 and found that the majority of their included studies (98 percent) proved the effectiveness of digital care visits in “improving patients’ overall health conditions and in assessing patients’ health conditions successfully” [71]. The same review found that more than a quarter of analyzed studies proved online consultations were as good as face-to-face visits [71]. However, digital care visits should not take over all in-person visits but act as a complement to the physical visits. It is important to note that the social interaction and physical presence facilitate better conditions for showing empathy and simply “being there” for the patient, which are essential parts of care and bear significant value to patients and professionals alike.

### 4.1. Implications of This Study

Admittedly, only a fraction of healthcare professionals’ specializations was included in the reviewed studies. The knowledge of the experience of surgeons, midwives, dental care professionals, and specialized physicians other than those included in this study is limited and should be studied in the future to get a clearer picture of their perspectives. The COVID-19 pandemic is surely transforming remote care, and there have already been studies that described the shift towards digital care visits and the organizational changes [11,18,72,73]. However, a more detailed review of how the perspectives have changed and how the rapid adoption has affected the use and experience of clinicians could be beneficial in the future. Moreover, more research could be done on the usability of digital care visits integrated into the EHR systems because, so far, the clinicians mostly use separate platforms. In addition, more explorations of how digital care visits could be combined with in-person care and the perspectives of professionals, patients, and caregivers on this approach could be studied further. Finally, more studies regarding the use of digital care visits in self-management and follow-up of chronically ill patients are needed. Knowing how digital care visits affect patient safety is also of interest to be studied in the future. Moreover, studying the economic impact of digital care visits on health care is also of great importance.

### 4.2. Strengths and Limitations

One of the strengths of this study is a comprehensive search strategy in three large databases containing large amounts of healthcare and technology-related publications. The search was carefully documented. The search queries were tested and adjusted to retrieve more relevant results. MeSH terms were used to broaden the search. Many studies were retrieved from the databases, and an additional search for the grey literature was performed. Various types of publications and different study types were included in the review to ensure broad coverage of the topic. All the citations were managed using Mendeley’s reference system to ensure orderly documentation.

This scoping review is not without limitations. Firstly, filtering the search results by the language (including only English papers) may have prevented getting more results and potentially missed data that could have been included in this study. Secondly, a limited time frame may have affected the quality and quantity of the findings. Additionally, lack of critical appraisal is one of the disadvantages of the scoping review type of studies and therefore poses a risk of bias [74]. The search terms and search queries were discussed in detail. The search queries were adjusted, and new search words were added several times. However, the screening and the selection of studies were performed by the first author, increasing the risk that some studies were missed [75].

## 5. Conclusions

To summarize, this scoping review explored the existing literature on the user experience of using digital care visits from different healthcare professionals’ points of view. The themes of positive experiences/benefits, facilitators, negative experiences/challenges, barriers, and suggestions for improvement were identified. The findings suggested that overall, healthcare professionals had a positive experience with the use of digital care visits and found numerous benefits of this type of remote care for themselves as healthcare workers as well as for their patients. Despite the overall positive experience, clinicians reported challenges and issues they faced when using the technology, including decision-making difficulties, physical barriers, technical issues, suitability concerns, and others. Finally, it is suggested that digital care visits cannot replace in-person visits in full. However, they could be effectively used in combination to treat and manage suitable conditions. Further research could be done to explore the experiences of other healthcare professionals not represented in this study, as well as the effects of the COVID-19 pandemic on digital care visit use.

## Figures and Tables

**Table 1:** Search strategy and the number of papers retrieved from the databases. The asterisk (*) in the search quies in PubMed and Web of Science represents any group of characters. It also represents no character.

Database	Search Words	Number of Papers
PubMed	(“digital visit*” [TIAB] OR “remote visit*” [TIAB] OR “remote consult*” [TIAB] OR teleconsultation [TIAB] OR “online consult*” [TIAB] OR “video consult*” [TIAB] OR videoconferencing [MH] OR videoconferencing [TIAB] OR “digital consult*” [TIAB] OR e-consultation* [TIAB] OR “electronic visit” [TIAB] OR “virtual visit” [TIAB]) AND (“medical professional*” [TIAB] OR “medical staff*” [MH] OR “medical staff*” [TIAB] OR “health personnel*” [TIAB] OR “health personnel*” [MH]) AND (experience* [TIAB] OR “user experience*” [TIAB] OR “user satisfaction” [TIAB])	n = 122
Web of Science	TS = (“digital visit*” OR “remote visit*” OR “remote consult*” OR teleconsultation OR “online consult*” OR “video consult*” OR “electronic visit*” OR “virtual visit*” OR “telemedicine*” OR “telehealth*” OR video conference* OR e-consult* OR e-health) AND TS = (“medical professional*” OR “medical staff*” OR “health* personnel” OR physician* OR nurs* OR therapist* OR midwi* OR “health* professional” OR “dentist*” OR “caregiver*” OR “pharmacist*”) AND TS = (experience* OR “user experience*” OR “user satisfaction”)	n = 1289
IEEE Xplore	((“All Metadata”: “digital visit” OR “All Metadata”: “remote visit” OR “All Metadata”: “remote consult” OR “All Metadata”: teleconsultation OR “All Metadata”: “online consult*” OR “All Metadata”: “video consult*” OR “All Metadata”: “electronic visit” OR “All Metadata”: “virtual visit” OR “All Metadata”: telemedicine OR “All Metadata”: telehealth OR “All Metadata”: videoconferenc* OR “All Metadata”: e-consult* OR “All Metadata”: e-health) AND (“All Metadata”: “medical professional” OR “All Metadata”: “medical staff” OR “All Metadata”: “health personnel” OR “All Metadata”: “health professional” OR “All Metadata”: physician OR “All Metadata”: nurs OR “All Metadata”: therapist OR “All Metadata”: midwi* OR “All Metadata”: dentist OR “All Metadata”: caregiver OR “All Metadata”: pharmacist) AND (“All Metadata”: experience* OR “All Metadata”: “user experience” OR “All Metadata”: “user satisfaction”))	n = 59

**Table 2:** General characteristics of the included studies.

Characteristics	Number of Studies	Reference Number
**Year of publication**		
2021	n = 13	[38,39,40,43,45,46,47,48,49,50,51,52,53]
2020	n = 9	[13,35,37,41,42,44,54,55,56]
2019	n = 2	[36,57]
2018	n = 1	[58]
2017	n = 2	[59,60]
2015	n = 1	[61]
**Country**		
Australia	n = 3	[36,49,59]
Belgium	n = 1	[42]
France	n = 1	[40]
Italy	n = 1	[54]
Norway	n = 2	[38,60]
Sweden	n = 3	[13,37,39]
The Netherlands	n = 1	[55]
USA	n = 14	[35,43,44,45,46,47,48,50,51,52,53,57,58,61]
Worldwide	n = 2	[41,56]
**Methodology/type**		
Qualitative study—semi-structured interviews	n = 5	[36,37,39,51,59]
Qualitative study—Focus groups	n = 1	[60]
Web-based survey	n = 12	[13,38,40,41,43,44,48,50,53,54,56,61]
Randomized controlled trial	n = 1	[58]
Descriptive study	n = 1	[52]
Observational survey	n = 1	[55]
Mixed methods study	n = 5	[35,45,46,47,57]
Design thinking—customer journey	n = 1	[42]
Review	n = 1	[49]
**Study participants**		
Mental health professionals	n = 8	[36,45,46,49,52,54,56,57]
Physicians	n = 15	[13,35,37,38,39,40,41,42,43,44,48,50,53,58,61]
Surgeons	n = 1	[55]
Nurses/nurse assistants/advanced practice professionals/residents/physical therapists/speech pathologists etc.	n = 8	[36,42,47,48,51,59,60,61]
Patients and/or caregivers	n = 7	[40,43,55,58,60,61]
Non-medical professionals (education staff, IT workers, social workers, care coordinators)	n = 4	[36,43,48,61]
**Studies related to COVID-19 pandemic**	n = 15	[35,36,40,41,42,43,44,45,46,47,48,49,50,52,54,56]

**Table 3:** Positive experiences/advantages and facilitators of the digital care visits use.

Theme	Category	Sub-Category	Reference
**Positive experiences/advantages**	Benefits of remote work	Flexible working hours and/or place	[13,37,39,45,46,54]
		Saved travel time/costs	[39,41,46]
		Feeling more relaxed and at ease	[39,54]
		Convenience	[45,46]
		Reduced workload	[37,39]
	Efficiency	Shorter visits	[13,47,48,50]
		Increased productivity/efficiency	[35,37,39,41,47,49,54,59]
	Satisfaction	Overall positive experience	[13,35,40,41,43,44,45,46,48,50,55,58,59]
		Easy to learn how to use	[41,44]
		Easy to use	[13,39,41,42,43,46,48,50,55,59,60,61]
		Satisfaction with the system/platform and/or its features	[13,46,47,58]
		Comfortable treating patients via digital care visits	[39,47]
		The interaction between healthcare professional and patient was satisfactory/effective	[39,43,48,55,58]
	Convenient, accessible care and saved resources for patients	Increased flexibility	[13,39,46,51,57,59]
		Greater accessibility	[13,39,41,46,51,52,58,59]
		Convenience	[45,58]
		Reduced costs and/or time for traveling	[13,46,51,52,57,58,59]
		Eliminated other costs	[51,57,58]
		Protection from communicable diseases	[39,51]
		Family inclusion and/or education	[51]
		Proper care for patients	[39,41,58]
	Patients‘ emotional state	Reduced stress, empowerment	[39,45,46,49,58,59]
		Confidence and increased cooperation	[45,46,59,60]
	Patient satisfaction	Satisfaction with digital care	[37,38,48,53]
**Facilitators**	New perspectives in remote care	Ability to get instant non-verbal feedback	[59]
		Ability to intervene in real-time	[57]
		Focusing on what is most important	[49,59]
		Less demanding	[39,59]
		Increased personal safety	[39,46]
		Observing themselves on video is helpful	[52]
		Insight into patient‘s home environment	[35,39,45,46,57]
		More frequent visits	[46,49,51]
		Continuity of care	[45,46]
		More personal visits	[59]
		Visits can be intimate	[52]
		Better than phone call consultations	[13,60]
	Technical qualities	Video and audio quality is acceptable/good	[13,37,38,43,48,55,59,61]
		No connectivity issues	[55]
	Possibilities of digital care visits	Possibility to consult/examine/diagnose/treat patients	[35,39,43,45,48,57,58,61]
		Possibility to work with patients’ emotions	[52]
		Possibility to build rapport with patients	[45,46]
		The relationship with patients was authentic	[48,56]
	Suitability	Suitable for delivering sensitive/bad news	[47]
		Suitable for follow-up visits	[38,40,54,55]
		Suitable to treat mental health problems	[38,39]
		Suitable for treating some skin conditions	[39,51]
		Suitable for administrative purposes	[38]
		Physical contact was not necessary	[40,55]
		Suitable for chronic disease management	[40]
		Suitable for palliative care	[35]
		Suitable for pediatric care	[35]

**Table 4:** Negative experiences/challenges and barriers of the digital care visits use.

Theme	Category	Sub-Category	Reference
**Negative experiences/Challenges**	Complicated decision making	Difficulties making decisions regarding patient’s diagnosis, treatment, or referrals	[13,37,38,39,46,50,55]
		Difficulties in guiding the right patients to digital care visits	[13,37,39]
		The need to rely on patient’s observations and descriptions	[35,39,51,52,59]
	Clinicians’ professional competence development	Concerns regarding loss of competence	[37]
		Lack of medical skills practice	[37]
	Work environment	Loneliness and isolation working from home	[37]
	Workload and workflow	Requires higher concentration	[45,54,57]
		More tiring	[45,49,54,56,57]
		Difficulties structuring time	[37,54]
		More stressful	[50]
		Administration or preparation takes time	[36,49]
		Lack of administrative support	[35,36,41]
	Dissatisfaction	Overall dissatisfaction with digital care visits	[53]
		Felt that patients’ needs were not adequately addressed	[53,60]
		Digital care visits are inferior to in-person visits	[35,45,50,52,53]
	Patient-professional relationship	Difficulty fostering rapport	[44,56]
		Difficulty in dealing with emotional situations	[45,49,52,56]
		Digital care visits are less personal	[52,60]
		Digital care visits were less intimate	[57]
		Difficulty in maintaining patient’s attention/engagement	[45,46,56,57]
	Unmet patients’ expectations	Patient’s desire for physical consultation was unmet	[51,60]
		Unrealistic patient expectations and poor understanding	[13]
		Patients are reluctant to pay for digital care visits	[51]
	Technical challenges	Patients lack technical skills	[45,51]
		Patients lack comfort in using technology	[45,49,51,57]
		Restricted access to technology due to socioeconomic status	[51]
	Complications from the patient’s side	More visit cancellations or rescheduling by patients	[57,59]
		Disruptions from patients’ side	[45,57]
	Patient safety and privacy	Safety concerns	[37,38,45]
		Privacy concerns	[45,52,56]
**Barriers**	Physical barriers	Inability to apply certain treatment techniques	[36,43,54]
		Inability to provide written information	[51]
		Lack of physical examination is problematic	[36,38,39,43,44,46,48,50,51,55,58,59,61]
		Inability to see non-verbal cues clearly	[45,48,49,50,52,57]
	Suitability	Inapplicable for some types of patients	[35,38,46,50,54]
		Inappropriate for sensitive conversations	[51]
	Technical issues	Connectivity issues	[36,42,45,46,50,52,53,58,60]
		Poor quality or lost audio and/or video	[42,46,52,53,58,60,61]
		Lack of technical support when working off office hours	[37]
		Lack of unified documentation system	[37]
		Difficult or uncomfortable to use	[35]
	Reimbursement issues	Ambiguity of insurance coverage status	[51]
		Training and administration time are not compensated	[36]
		Reimbursement model needs to be adapted	[13,41,44]

**Table 5:** Suggestions for improvement.

Theme	Category	Sub-Category	Reference
**Suggestions for improvement**	Training and education	Provide proper training in using the technology	[13,36,42,44,47,56]
		Tutorial materials on how to use the technology for professionals and/or patients	[55,59]
		Promotion and education on digital care visits	[41,44]
	System and tools	Standardized equipment for providers	[47]
		Incorporate video-conferencing tools into the EHR system	[37]
		Implement triage system	[13,37,50]
		Enhanced data security	[42]
		Use double web-cameras	[49]
	Clinician support	Promotion of self-care for healthcare professionals	[46]
		Incorporate administration/coordination support	[42,44,47]
		Ensure access to a suitable work environment and tools	[45]

## Data Availability

All relevant data are included in the article.
